# Commentary on “Accelerated biological aging and postoperative acute kidney injury in surgical patients”

**DOI:** 10.1097/JS9.0000000000004743

**Published:** 2026-01-21

**Authors:** Huiwen Luo, Lefeng Tian, Deng Hu, Yuanqiao Teng, Honggang Shao, Haili Long

**Affiliations:** a NHC Key Laboratory of Nuclear Technology Medical Transformation, Mianyang Central Hospital, School of Medicine, University of Electronic Science and Technology of China, Mianyang, China; b Department of Operating Room, Mianyang Central Hospital, School of Medicine, University of Electronic Science and Technology of China, Mianyang, China; c Department of Urology, Mianyang Central Hospital, School of Medicine, University of Electronic Science and Technology of China, Mianyang, China

*Dear Editor,*

The study by Yin et al. provides compelling evidence that accelerated biological aging, quantified using Phenotypic Age Acceleration (PhenoAgeAccel), is independently associated with postoperative acute kidney injury (AKI) and severe AKI among 94 006 surgical patients. This work expands the growing recognition that biological age, rather than chronological age, more accurately captures an individual’s physiological vulnerability and heterogeneity in aging processes^[1,2]^.

A major strength of this study lies in its scale and methodological rigor. The multicenter design, combined with detailed perioperative data and standardized AKI assessment, allows robust evaluation of the association between biological aging and postoperative renal outcomes. Importantly, PhenoAgeAccel demonstrated markedly better discriminatory power than chronological age for predicting AKI and AKI stage 2 +, consistent with recent studies showing the superior prognostic utility of biomarker-based biological age markers across cardiovascular, renal, and neurocognitive domains^[3-5]^.

The observed dose–response relationship further reinforces the biological plausibility of this association: patients with the highest acceleration of biological age exhibited more than threefold higher adjusted hazards of AKI and nearly tenfold higher hazards of severe AKI compared with biologically younger peers. These findings align with mechanistic evidence indicating that accelerated biological aging is accompanied by mitochondrial dysfunction, impaired repair capacity, chronic low-grade inflammation, and cellular senescence – all of which exacerbate susceptibility to perioperative organ injury^[6,7]^.

Despite its strengths, several considerations merit discussion. First, the PhenoAge formula incorporates serum creatinine, which also forms the basis of AKI diagnosis. Although sensitivity analyses excluding creatinine yielded consistent associations, partial collinearity cannot be entirely ruled out. Future work may consider creatinine-independent aging metrics, such as epigenetic clocks^[8]^. Second, granular perioperative physiological data – such as vasopressor dose, cumulative hypotension time, urine output, and fluid balance – were not available. These factors are known to significantly influence postoperative AKI risk and may partially mediate the relationship between biological aging and renal outcomes^[9]^. Third, because PhenoAgeAccel captures systemic inflammation, nutritional status, hematologic variability, and multi-organ reserve, it remains unclear whether accelerated biological aging is an independent biological construct or a composite surrogate for multimorbidity and frailty. Integrating biological aging with validated frailty assessments may enhance predictive accuracy^[10]^.

Nevertheless, the study’s findings carry important clinical implications. Identification of accelerated biological aging preoperatively could support tailored perioperative risk stratification and optimization strategies, potentially including prehabilitation interventions, enhanced hemodynamic monitoring, avoidance of nephrotoxic exposures, and individualized anesthesia planning. Whether interventions that modify biological age can improve surgical outcomes represents a promising area for prospective research.

In summary, Yin *et al* present high-quality evidence that accelerated biological aging is a significant predictor of postoperative AKI and severe AKI, surpassing traditional chronological age in discriminatory capacity. This study represents an important step toward incorporating biological aging biomarkers into precision perioperative risk assessment and highlights an innovative direction for future research.

## Data Availability

All data analyzed or generated during this study are included in the article.

## References

[1] Kivimäki M Frank P Pentti J. Proteomic organ-specific ageing signatures and risk of age-related diseases. Lancet Digit Health 2025;7:e195–e204. 40015764 10.1016/j.landig.2025.01.006

[2] Huang Y Da L Dong Y. Construction and validation of a DNN-based biological age in China Kadoorie biobank. GeroScience 2025;47:4241–52. 40050560 10.1007/s11357-025-01577-x PMC12181170

[3] Dai Q Sun H Yang X. Clinical biomarker-based biological age and cardiovascular risk in Chinese adults. BMC Public Health 2025;25:868. 40038610 10.1186/s12889-025-22114-7 PMC11881332

[4] Gao X Geng T Jiang M. Accelerated biological aging and risk of depression and anxiety: evidence from 424,299 UK Biobank participants. Nat Commun 2023;14:2277. 37080981 10.1038/s41467-023-38013-7 PMC10119095

[5] Zheng G Chang Q Zhang Y. Associations of clinical parameter-based accelerated aging, genetic predisposition with risk of chronic kidney disease and associated life expectancy: a prospective cohort study. Aging Cell 2024;e14453. 39717880 10.1111/acel.14453 PMC11984662

[6] Rex N Melk A Schmitt R. Cellular senescence and kidney aging. Clin Sci (Lond) 2023;137:1805–21. 38126209 10.1042/CS20230140 PMC10739085

[7] Li X Li C Zhang W. Inflammation and aging: signaling pathways and therapies. Signal Transduct Target Ther 2023;8:239. 37291105 10.1038/s41392-023-01502-8 PMC10248351

[8] Kivimäki M Frank P Pentti J. Proteomic organ-specific ageing signatures and 20-year risk of age-related diseases: the whitehall II observational cohort study. Lancet Digit Health 2025;7:e195–e204. 40015764 10.1016/j.landig.2025.01.006

[9] Cheruku SR Raphael J Neyra JA Fox AA. Acute kidney injury after cardiac surgery: prediction, prevention, and management. Anesthesiology 2023;139:880–98. 37812758 10.1097/ALN.0000000000004734 PMC10841304

[10] Wang SK Wang QJ Wang P. The impact of frailty on clinical outcomes of older patients undergoing enhanced recovery after lumbar fusion surgery: a prospective cohort study. Int J Surg 2024;110:4785–95. 38729123 10.1097/JS9.0000000000001594 PMC11325916

